# Seasonality and Differential Growth Patterns of Body Dimensions of Children in a Rural Community of Yucatan, Mexico

**DOI:** 10.1002/ajhb.70015

**Published:** 2025-03-12

**Authors:** Lucely Nataly Molina‐Félix, Barry Bogin, Sudip Datta Banik

**Affiliations:** ^1^ Laboratory of Neuroscience, Center for Applied Public Health Research, Faculty of Medicine, Autonomous University of Sinaloa Culiacan Sinaloa Mexico; ^2^ Emeritus Professor of Biological Anthropology, School of Sport, Exercise and Health Sciences, Loughborough University, Loughborough, United Kingdom; Member UCSD/Salk Center for Academic Research and Training in Anthropogeny (CARTA) San Diego California USA; ^3^ Department of Human Ecology Centro de Investigación y de Estudios Avanzados del Instituto Politécnico Nacional (Cinvestav‐IPN) Merida Yucatan Mexico

**Keywords:** body dimensions, knee height, preschool children, seasonality, sitting height

## Abstract

**Background:**

Seasonality of human growth evinces the association between environmental variation, including the physical and the social–economic–political environment, and biological changes. The objective of the present study was to evaluate the seasonality of the growth of body dimensions (absolute and relative to height) of 2‐ to 5‐year‐old children and their differential increment (percentage changes) in the dry, rainy, and “*nortes*” seasons at Quintana Roo in Yucatan, Mexico.

**Methods:**

The study was mixed‐longitudinal. Repeated anthropometric measurements (height, weight, head circumference, sitting height, knee height) were recorded at the end of the dry, rainy, *nortes*, and again dry seasons of 31 preschool children in Quintana Roo, Yucatan. The derived variables (body dimensions relative to height) were the sitting height ratio (SHR) and knee height ratio (KHR). Data on seasonal variation in children's eating habits, availability of food items, and frequencies of signs, symptoms, and illnesses reported by the mothers were recorded.

**Results:**

The participants grew more in height and other body lengths between the *nortes* and dry seasons. Body weight increased most during the *nortes* and least in the rainy season when the frequencies and duration of illness were higher. Differences in mean values between the seasons were higher for KHR than for SHR. Children's eating habits, the availability of food items, and the frequencies of signs and symptoms of illness were different in the seasons.

**Conclusion:**

Seasonality and differential growth patterns of body dimensions were observed in preschool children. The growth of the lower leg length (knee height) was more sensitive to seasonality than the trunk (sitting height).

## 1 Introduction

Seasonality profoundly affects a wide range of activities and biological processes in different species, including humans. It represents a regular pattern or variation correlated with the astronomical, meteorological, and biophysical rhythms of the ecosystems, populations, and individuals who live therein. Seasonality is essential for understanding the interrelationships between human beings and the natural and social environments where they belong (Devereux et al. 2012; Ulijaszek and Strickland 1993).

Historically, seasonal variations in the physical and sociocultural environment have shaped different ways and means of life, causing changes that directly or indirectly influence human biology and behavior and the dynamics of the life of the individuals in a population, demanding them to cope with those factors (Ulijaszek and Strickland 1993). In this regard, given the sensitivity and close relationship of human beings with their ecosystems, seasonal variations produce changes not only in the climate but also in related patterns such as workload, food production, food intake, diseases, and child growth (Devereux et al. 2012; Ulijaszek and Strickland 1993).

The first written findings of a seasonal variation in growth rate were published by Buffon 1777 in his encyclopedia *Histoire Naturelle*, where he studied the growth records of the son of the Count of Montbéliard, who measured his son's height every 6 months from birth to the age of 18 and noted that each year most of the boy's increase in height took place during the spring and summer months (Buffon 1777). Since then, many studies have reported seasonal variations in the child growth rate in other populations (reviewed in Bogin 2021).

The effects of seasonality on behavior and health patterns and their consequences for human biology may vary across populations, as they depend mainly on individual and community coping resources. In agricultural and rural areas, especially in tropical countries, seasonality shapes ways of life and livelihoods in profound but often negative ways, with potential biological outcomes on growth and morbidity (Devereux et al. 2012). The timing of the rainy season usually causes changes in food availability and the incidence of diseases, which consequently leads to a growth deficit, with lower height and weight gains during the rainy season compared to those in the dry season. Evidence for this cause of seasonal growth changes was gathered mainly in Africa and Asia (Billewicz and McGregor 1982; Hauspie and Pagezy 1989; Hillbruner and Egan 2008; Mohsena et al. 2018; Panter‐Brick 1997; Spencer et al. 2017; Tomkins et al. 1986).

On the other hand, in the countries of temperate regions and groups of people with a high socioeconomic level who have more significant resources to deal with the adverse effects of seasonality on resource availability and exposure to environmental stressors, the causes of seasonal variation in growth rate have been associated with other factors such as day length and exposure to sunlight, temperature, physical activity, and dietary preferences at different times of the year (Bogin 2021). Evidence for this cause of seasonal growth changes was gathered mainly in the countries of the American continent (Bogin 1978; Reynolds and Sontag 1944), Europe (Cole 1993; Dalskov et al. 2016; Gelander et al. 1994; Marshall 1971; Tillmann et al. 1998), and Asia (Xu et al. 2001).

Overall, these studies reported more significant height gains in spring and summer (or in the dry season) and more substantial weight gains in the autumn and winter (or rainy season) months. Noteworthy is the observed lag of one or more months between the most rapid increases in height and weight. Conjointly, the findings reported in the literature reveal the enormous sensitivity of human beings to their seasonal environmental variations, particularly during the first years of life, as well as the influence of these factors on the diversity of human growth patterns.

Human growth is a continuous process starting from the prenatal stage when maternal capital (biological as well as psychosocial and economic) significantly impacts fetal growth (Wells 2010). In addition to the maternal life history trade‐off and intergenerational influences on offspring growth and development, early‐life environmental factors may have a long‐term impact on growth and maturation in late childhood and adolescence and the development of chronic diseases in adulthood (Wells 2011).

Height and weight are the most elementary measurements used to evaluate human growth. However, there are others, such as circumferences and lengths of body dimensions, which, altogether, provide information for assessing physical growth patterns in children and adolescents (Bogin 2021). Biologically, each of these measurements reflects different aspects of growth. Stature is a linear and cumulative measure usually used to assess long‐term changes in body dimensionality resulting from changes in the length of the upper and lower body segments (Gordon et al. 1988). From birth to adulthood, stature increases with time, but the rate of increase varies according to many factors (genomic regulation, endocrine production, nutrition, health, etc.). Weight is usually used to assess the relatively short‐term changes in total body mass; it includes lean and fat mass and reflects the growth of all body tissues (Hauspie et al. 2004). Weight may increase or decrease from birth to adulthood due to many factors. Leg length and leg length relative to height have been studied because these are sensitive “biomarkers” of early‐life environmental conditions (Gunnell 2002; Bogin and Varela‐Silva 2010).

Considering the above and the relative paucity of information on the seasonal variation of the growth of body dimensions in infancy and early childhood, we were interested in researching the seasonality of human growth in a rural community of Yucatan, Mexico, where people had relatively homogeneous household socioeconomic status (SES) backgrounds. We recorded the relative consumption of different food items by the preschool children and the signs and symptoms of illness reported by the mothers in three seasons recognized by the community: dry, rainy, and *nortes* (northerly cold fronts). In this regard, the present study aimed to evaluate the seasonality of the growth of body dimensions (absolute and relative to height) of 2‐ to 5‐year‐old children and their differential increment (percentage changes) in the mentioned seasons at Quintana Roo, Yucatan, Mexico.

## 2 Methods

### 2.1 Sociodemography of the Population

**FIGURE 1:**
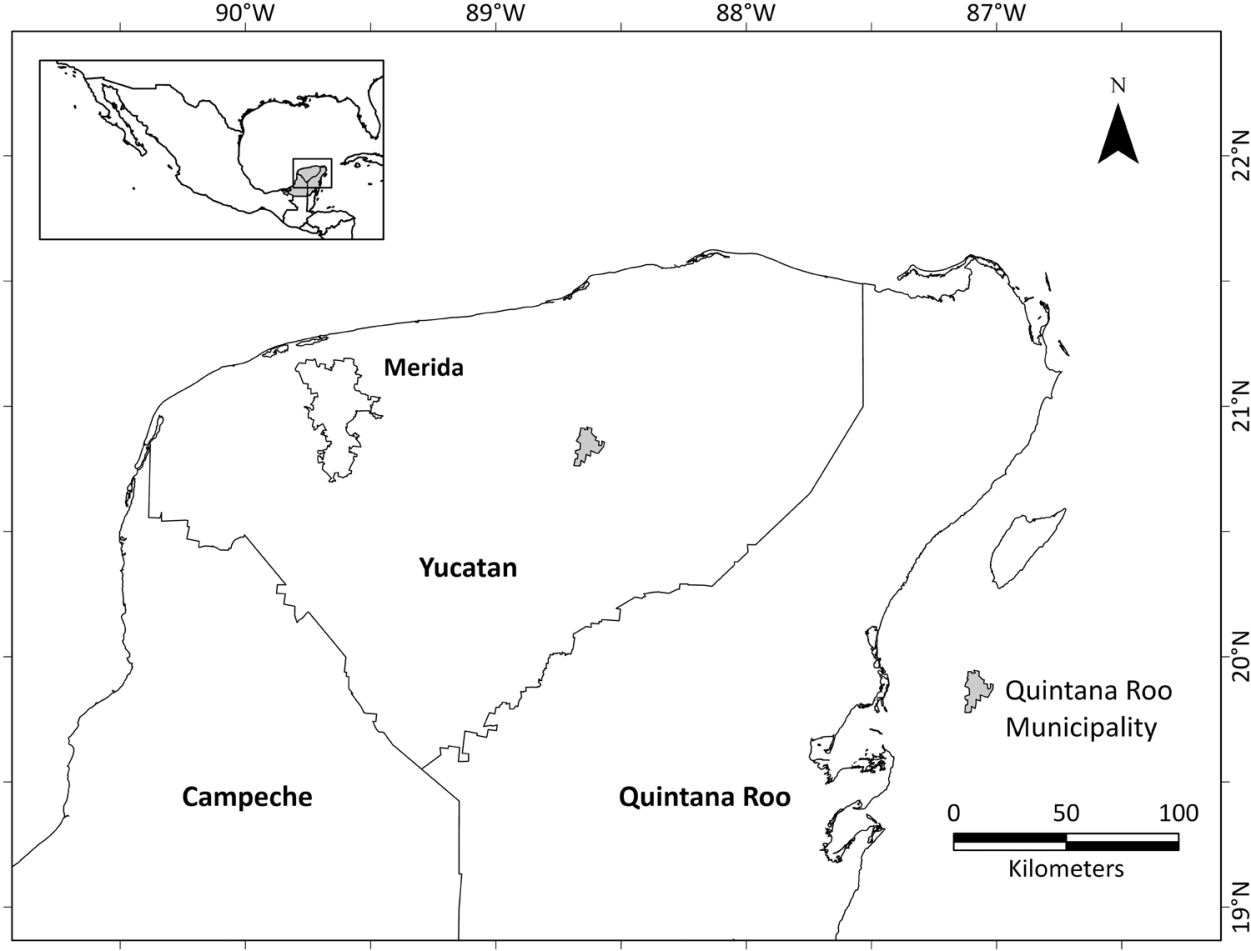
Study location: Quintana Roo municipality in Yucatan, Mexico.

The study was carried out at Quintana Roo village, in Yucatan, Mexico, located approximately 115 km southeast of the city of Merida, the state capital (Figure 1, the village of Quintana Roo is not to be confused with the State of Quintana Roo). This village has the smallest population size among the 106 municipalities in Yucatan; 976 inhabitants with Mayan ancestry live in the 305 households; the majority have electricity, drainage, sanitary services, and access to government health services (97.4%, 88.8%, 87.5%, and 86.1%, respectively) (INEGI 2021a). A previous survey (López‐Moreno 2021) connected with the same research project, “Integrated Community Food and Nutrition Project in Yucatan,” reported an overall homogeneous household SES (level of education, occupation, monthly per capita income expenditure for food and other items) based on the established criteria by the Mexican Government's official guidelines (INEGI 2011, 2021b). In the present study, we also observed similar household SES indicators, including parents' level of education and occupation. Most fathers were engaged in agricultural activities, while mothers were involved in household activities. A large part of family income came from social programs; 70.97% of participating families were affiliated with one.

### 2.2 Agricultural Production System of the Region of Study

The most important local agricultural production system is known as “*milpa*,” a polyculture composed of varieties of plants such as corn, beans, and squash as the main crops, as well as roots and tubers. Other activities such as hunting, apiculture, and using forest resources are linked to this productive system, which provides food, firewood, fodder, building materials, instruments, utensils, and medicinal plants (Terán and Rasmussen 2009). Larger *milpas* are often located relatively close to the community, on fields of 1–3 ha (10 000–30 000 m^2^) (Santos‐Fita et al. 2013).

In addition, backyard gardens, locally known as *patio* or *solar*, are intimate spaces close to the home intended for plant management that require irrigation and raising animals under a domestic group's guidance, especially women. They are considered part of the productive system of the *milpa* and offer complementary food resources such as fruits, vegetables, spices, and animal protein (Lope‐Alzina 2017; Terán and Rasmussen 2009). Edible plants predominate in the cultivation of plants in the *patio* or *solar*, mainly fruit trees, while in animal husbandry, the most common species are chickens, turkeys, pigs, and ducks. The inhabitants often exchange these resources for other products or money (Terán and Rasmussen 2009). The diversity of species in backyards in the Yucatan Peninsula and their multifunctionality are attributes that make them remarkable in Mesoamerica (Lope‐Alzina 2017), with positive contributions of the diversity of these spaces to household food security in different communities in Yucatan (Castañeda‐Navarrete 2021).

In the community of Quintana Roo and different rural communities of the region, another space that expands the agricultural production system is the community plots where the Mexican Government executes the social development program *“Sembrando Vida”* (“Planting Life”, in English). A program that grants economic support to the affiliated farmers for planting, caring for, and harvesting traditional crops and fruits, and wood species, some of which can be transplanted to their *milpa* and *patio* or *solar*. In this sense, different productive spaces in the same agricultural cycle increase the possibility of obtaining a harvest (Terán and Rasmussen 2009).

An important aspect to highlight in the contextualization of the study region is that although the extensive local production system constitutes an essential part of the food base, currently, in most rural communities of Yucatan, including Quintana Roo, the primary source of food comes from local stores, subsidies, and markets outside the community (Castañeda‐Navarrete 2021; Gurri et al. 2021). This ease of access to processed foods has led to changes in the traditional diet, from a diet based on a variety of locally grown foods to a diet based on highly processed foods, rich in fats, sugars, and low in fiber (Bogin et al. 2014).

### 2.3 Climate and Seasons of the Region of Study

The Yucatan Peninsula is characterized by three seasons: the dry season (March to June), with scarce precipitation (0–30 mm) and high temperatures (36°C–38°C); the rainy season (July to October), distinguished for having the maximum precipitation (220 mm) and temperature (38°C) within the year; and the “*nortes*” season (November to February), a period of marginal precipitation (40 mm) and low temperatures (23°C), influenced by northerly cold fronts of polar air (CONAGUA 2021; Medina‐Gómez and Herrera‐Silveira 2009). The climate in the region of Quintana Roo, Yucatan, is subhumid warm with rain in the rainy season, with an average temperature between 26°C and 28°C and an annual precipitation rate between 1000 and 1200 mm (INEGI 2010).

### 2.4 Study Design

The present study was descriptive and mixed‐longitudinal in nature. The sample included 2.00‐ to 5.99‐year‐old children (*n* = 31). To estimate the seasonal variation of the physical growth of children, we recorded anthropometric measurements in June 2021 (end of the dry season), October 2021 (end of the rainy season), February 2022 (end of the *nortes* season), and June 2022 (end of the dry season).

Fieldwork started in March 2021. The community health center provided a census of children. We identified households, and the parents and caregivers of 2‐ to 5‐year‐old children were invited to participate and informed about the project's aims. Of the 47 children of this age group living in the community, 42 parents agreed to participate and signed the informed consent form; we took verbal permission from the participating children. However, during the fieldwork, 11 of the participants could not continue in the project due to their health conditions or not being available; unfortunately, one of the participants died. The final sample size was 31 children (12 boys and 19 girls) (Table 1).

**TABLE 1:** Sample size by age and sex of 2‐ to 5‐year‐old children (*n* = 31) of Quintana Roo, Yucatan.

Age (years)	Boys *n*	Girls *n*	Total *n*
2	1	3	4
3	6	5	11
4	2	5	7
5	3	6	9
Total	12	19	31

### 2.5 Variables

#### 2.5.1 Anthropometry

We recorded anthropometric measurements of height (cm), weight (kg), head circumference (HC) (cm), sitting height (SH) (cm), and knee height (KH) (cm) following a standard protocol (Lohman et al. 1988). We measured height (cm) using a portable stadiometer (Seca, Germany) with a precision level of 0.1 cm and weight (kg) using an electronic scale (Omron, Japan) with a precision level of 0.05 kg. We used a standard non‐elastic tape (Lufkin, USA) to measure HC, the first segment of a Martin‐type anthropometer for SH and KH measurements, and a standard anthropometric bench (30 × 50 cm and height 40 cm) for measuring SH.

We calculated two derived variables: sitting height ratio [(SHR = sitting height/height) × 100] and knee height ratio [(KHR = knee height/height) × 100]. These variables estimated differential growth patterns of body size (absolute and relative to height). We used anthropometric characteristics of the participating children to determine the percentage change, increase or decrease, between the three seasons. To calculate the percentage, we subtracted the value of the measurement of the last month (*X*2) from the measurement of the first month (*X*1) of the corresponding season. Subsequently, this result was divided by the measurement value of the first month of that season (*X*1) and multiplied by 100 to obtain the percentage change per season. The equation was exemplified as follows: [(*X*2−*X*1)/*X*1] × 100 = percentage change by season.

#### 2.5.2 Children's Eating Habits, Availability of Food Items, and Signs, Symptoms, and Illness

In the present study, we used data on seasonal variation in children's eating habits, availability of food items, and frequencies of signs, symptoms, and illnesses reported by the mothers and caregivers as the correlates of seasonal variation of physical growth of children.

We collected information on children's eating habits through a questionnaire that included questions focused on assessing the main food components of the diet and their availability. A pre‐validated questionnaire (López‐Moreno 2021) was used based on fieldwork experience in the study community; it included the most consumed traditional and processed foods and some regularly consumed items within the age range of interest reported in the literature. With this information, we adapted a food consumption frequency questionnaire. We investigated the frequency of consumption 7 days before applying the questionnaire and its origin (purchase, own production, borrowed, gifted, food aid). We examined the output of cultivable foods or animal husbandry in depth, the place destined for this activity within the local agricultural production system (*milpa*, *patio* or *solar*, and plots), and the foods grown and harvested in each area.

We collected health information using a pre‐validated questionnaire (used in the studies undertaken in this region previously by SDB, one of the co‐authors) that listed a series of signs, symptoms, and illnesses focused mainly on problems of the respiratory and digestive systems: flu, cough, vomiting, diarrhea, and gastrointestinal infections, among others. We asked the mothers and caregivers about the presence of these signs, symptoms, and illnesses in children in the month before the end of a particular season and their frequency and duration. Additionally, we recorded the type of assistance received and the type of medical service attended in cases where illness was confirmed. We obtained the data in June 2021, October 2021, February 2022, and June 2022, corresponding to the last month of the dry, rainy, *nortes*, and again dry seasons, respectively.

Another survey (López‐Moreno 2021), carried out before the present study, assessed the perception of household food insecurity (HFI) as a proxy of the SES of the inhabitants of Quintana Roo, Yucatan. The survey recorded HFI using the Latin American and Caribbean Food Security Scale (ELCSA in Spanish acronym) (Segall‐Correa et al. 2012). The response to this survey was 219 out of the total 221 households (INEGI 2011). Based on these results, we defined the HFI as a proxy variable to evaluate the SES of the households participating in this study. Data on parents' age, education, occupation, monthly per capita household income, and food expenditure were collected. The household crowding index was calculated using information on the number of rooms and the number of members living in the house; the overcrowding index was obtained when the average number of occupants per bedroom was greater than 2.5 people (INEGI 2017).

### 2.6 Data Analysis

Data analysis was done using the Statistical Package for the Social Sciences program (SPSS version 19.0). We performed further analysis of eating habits. According to the Mexican system of food classification, we classified foods into eight different groups (cereals, legumes, vegetables, fruits, animal‐origin foods, dairy products, fats, and sugars) (Pérez‐Lizaur et al. 2014). Subsequently, based on the frequency of consumption, we calculated the relative frequency of weekly consumption by food groups and the average of the entire sample to determine the frequency of consumption of each food group relative to the total consumption of food items. Based on the reports, the frequency and duration of signs, symptoms, and illness, and the frequency of food production were calculated.

Regarding growth, we calculated descriptive statistics (mean and standard deviation values) and percentage changes of the measurements (absolute and relative to height) from one season to the following. Anthropometric measurements were recorded by a single researcher (LNMF); technical errors of measurement (TEM) were calculated on the measurements taken from 10 randomly selected children, and the errors were found to be within reference values (Ulijaszek and Kerr 1999). The coefficient of reliability (%) values were > 95.0, indicating measurement precision (Androutsos et al. 2020; Stomfai et al. 2011). Anthropometric variables were normally distributed following the criteria of the Shapiro–Wilk test (*p* > 0.05). Height (cm), body weight (BW) (kg), SH (cm), and KH (cm) of children were transformed into *z*‐score values (height‐for‐age, weight‐for‐age, sitting height‐for‐age, and knee height‐for‐age) using the World Health Organization (WHO) growth reference (Frisancho 2008).

We tested the significance of differences in mean values of the measurements between two consecutive seasons using a paired *t*‐test. A repeated measures ANOVA (general linear model) was run to understand the significant differences in mean values of height and other measurements recorded at the end of dry, rainy, *nortes*, and dry seasons in 2021–2022. The model was adjusted for age and sex (male and female). In all tests, we set statistical significance at *p* < 0.05.

## 3 Results

### 3.1 Household SES Background (a Brief Description of the Sample)

The mother's mean age was 32.19 years (minimum 19 and maximum 42 years) in June 2021. Parent's education data showed that 48.38% of mothers and 40% of fathers had secondary education; others had an incomplete high school education (35.47% mothers, 40% fathers) and only one of both sexes had a university education (3.23% and 4%, respectively). Most fathers were agriculturists (68%), and the rest (32%) were service holders in the public and private sectors. Mothers were mostly housewives (45.16%), 25.81% were engaged in the sale of prepared foods or worked as employees in local businesses, and 12.9% worked as domestic employees. The reported average per capita monthly household income was 120 USD, and the monthly food expenditure was 80 USD. People lived in tiny houses, and 58.1% of households had a high overcrowding index (2.5 individuals or more living in a single room).

The prevalence of HFI in the community, as previously recorded by López‐Moreno (2021) from a census of 219 households, was quite notable; 6% reported severe, 16% moderate, and 56% low HFI. Frequencies were estimated in the sample of 31 households of the present study: 19.2% did not perceive food insecurity, whereas 50% perceived low, and 30.8% perceived moderate HFI.

### 3.2 Anthropometry

Mean values of age of the total sample of 2‐ to 5‐year‐old children during the survey were 3.65, 3.89, 4.23, and 4.56 years in June 2021, October 2021, February 2022, and June 2022, respectively. We did not observe significant sex differences in the mean values of anthropometric characteristics; therefore, we presented the results using the pooled sample of children (*n* = 31).

Mean height value at the beginning of the seasonal cycle was 95.48 and 102.30 cm at the end. These values were 15.52 and 17.64 kg for BW, 48.83 and 50.02 cm for HC, 54.05 and 57.21 cm for SH, and 28.15 and 30.54 cm for KH, respectively. This reflects cumulative increases in body size and mass during this period. Across all seasons, SH was 56% of standing height, followed by KH (29%). As children grew older, we observed that the SHR decreased while the KHR increased. The mean values of SHR did not show a significant increase in the rainy (difference of values between the end of June and the end of October 2021), the *nortes* (difference of values between the end of October 2021 and the end of February 2022), and the dry seasons (difference of values between the end of February and the end of June 2022). However, KH showed significant differences in mean values between two consecutive seasons (Table 2).

**TABLE 2:** Descriptive statistics of anthropometric characteristics (absolute and relative dimensions) across the three seasons in 2‐ to 5‐year‐old children (*n* = 31) of Quintana Roo, Yucatan.

Variables	A	B	C	D	Pairwise comparisons [mean difference (paired *t*, *p*; 95% CI values)]		
	Mean (SD)	Mean (SD)	Mean (SD)	Mean (SD)	A and B	B and C	C and D
Height (cm)	95.48 (8.40)	97.95 (8.17)	100.48 (7.89)	102.30 (7.68)	−2.47 (−14.53, ***p* < 0.0001**; CI: −2.82, −2.13)	−2.53 (−13.58, ***p*** **< 0.0001**; CI: −2.91, −2.15)	−1.82 (−13.37, ***p* < 0.0001**; CI: −2.10, −1.54)
HAZ	−0.58 (1.07)	−0.44 (1.24)	−0.48 (1.13)	−0.54 (1.18)	−0.01 (−0.10, *p* = 0.92; CI: −0.22, 0.2)	0.06 (0.7, *p* = 0.48; CI: −0.11, 0.22)	0.06 (0.75, *p* = 0.458; −0.11, 0.23)
BW (kg)	15.52 (3.74)	15.94 (3.91)	17.10 (4.06)	17.64 (4.18)	−0.42 (−3.22, ***p* = 0.003**; CI: −0.69, −0.15)	−1.16 (−7.73, ***p* < 0.0001**; CI: −1.46, −0.85)	−0.54 (−4.10, ***p* < 0.0001**; CI: −0.8, −0.27)
WAZ	0.38 (1.07)	−0.44 (1.29)	0.57 (1.30)	0.48 (1.30)	0.08 (1.18, *p* = 0.248; CI: −0.06, 0.22)	−0.14 (−1.96, *p* = 0.06; CI: −0.29, 0.01)	0.09 (1.78, *p* = 0.085; CI: −0.01, 0.19)
HC (cm)	48.83 (1.58)	49.23 (1.57)	49.48 (1.55)	50.02 (1.54)	−0.40 (−7.26, ***p* < 0.0001**; CI: −0.51, −0.29)	−0.25 (−5.40, ***p* < 0.0001**; CI: −0.35, −0.16)	−0.54 (−10.32, ***p* < 0.0001**; CI: −0.65, −0.43)
SH (cm)	54.05 (3.6)	55.18 (3.42)	56.37 (3.51)	57.21 (3.63)	−1.13 (−8.39, ***p* < 0.0001**; CI: −1.40, −0.85)	−1.19 (−9.55, ***p* < 0.0001**; CI: −1.44, −0.94)	−0.84 (−12.34, ***p* < 0.0001**; −0.98, −0.70)
SHZ	−0.13 (0.96)	0.06 (1.10)	0.14 (1.09)	0.09 (1.15)	−0.05 (−0.76, *p* = 0.454; CI: −0.20, 0.09)	−0.10 (−1.74, *p* = 0.093; CI: −0.22, 0.02)	0.05 (0.97, *p* = 0.337; CI: −0.05, 0.15)
SHR (%)	56.73 (1.93)	56.45 (1.93)	56.19 (1.77)	55.98 (1.64)	0.28 (2.62, *p* = 0.14; CI: 0.06, 0.05)	0.26 (1.73, *p* = 0.95; CI: −0.05, 0.57)	0.19 (1.76, *p* = 0.88; CI: −0.03, 0.43)
KH (cm)	28.15 (2.90)	28.90 (2.89)	29.80 (2.75)	30.54 (2.77)	−0.75 (−8.51, ***p* < 0.0001**; CI: −0.93, −0.57)	−0.90 (−13.33, ***p* < 0.0001**; CI: −1.03, −0.76)	−0.74 (−10.12, ***p* < 0.0001**; CI: −0.89, −0.59)
KHZ	2.09 (0.53)	2.18 (0.56)	2.19 (0.54)	2.18 (0.58)	−0.03 (−0.60. *p* = 0.55; CI: −0.14, 0.08)	−0.01 (−0.30, *p* = 0.77; CI: −0.11, 0.08)	0.01 (0.29, *p* = 0.77; CI: −0.06, 0.08)
KHR (%)	29.45 (0.76)	29.47 (0.70)	29.63 (0.67)	29.83 (0.77)	−0.02 (−0.20, *p* = 0.84; CI: −0.19, 0.16)	−0.16 (−2.37, ***p* = 0.02**; CI: −0.3, −0.2)	−0.20 (2.25, ***p* = 0.03**; CI: −0.38, −0.02)

*Note:* A: June 2021 (end of the dry season); B: October 2021 (end of the rainy season); C: February 2022 (end of the *nortes* season); D: June 2022 (end of the dry season). Bold values = *p* < 0.05.

Abbreviations: BW, body weight; CI, confidence interval; HAZ, height‐for‐age *z*‐score; HC, head circumference; KH, knee height; KHR, knee height ratio; KHZ, knee height‐for‐age *z*‐score; SD, standard deviation; SH, sitting height; SHR, sitting height ratio; SHZ, sitting height‐for‐age *z*‐score; WAZ, weight‐for‐age *z*‐score.

*Z*‐score values of height and weight‐for‐age were within normal limits; the same with SH and KH. A paired *t*‐test of the *z*‐score values between two successive seasons did not show significant differences in mean values (results are presented with mean difference and 95% confidence interval values) (Table 2).

**FIGURE 2:**
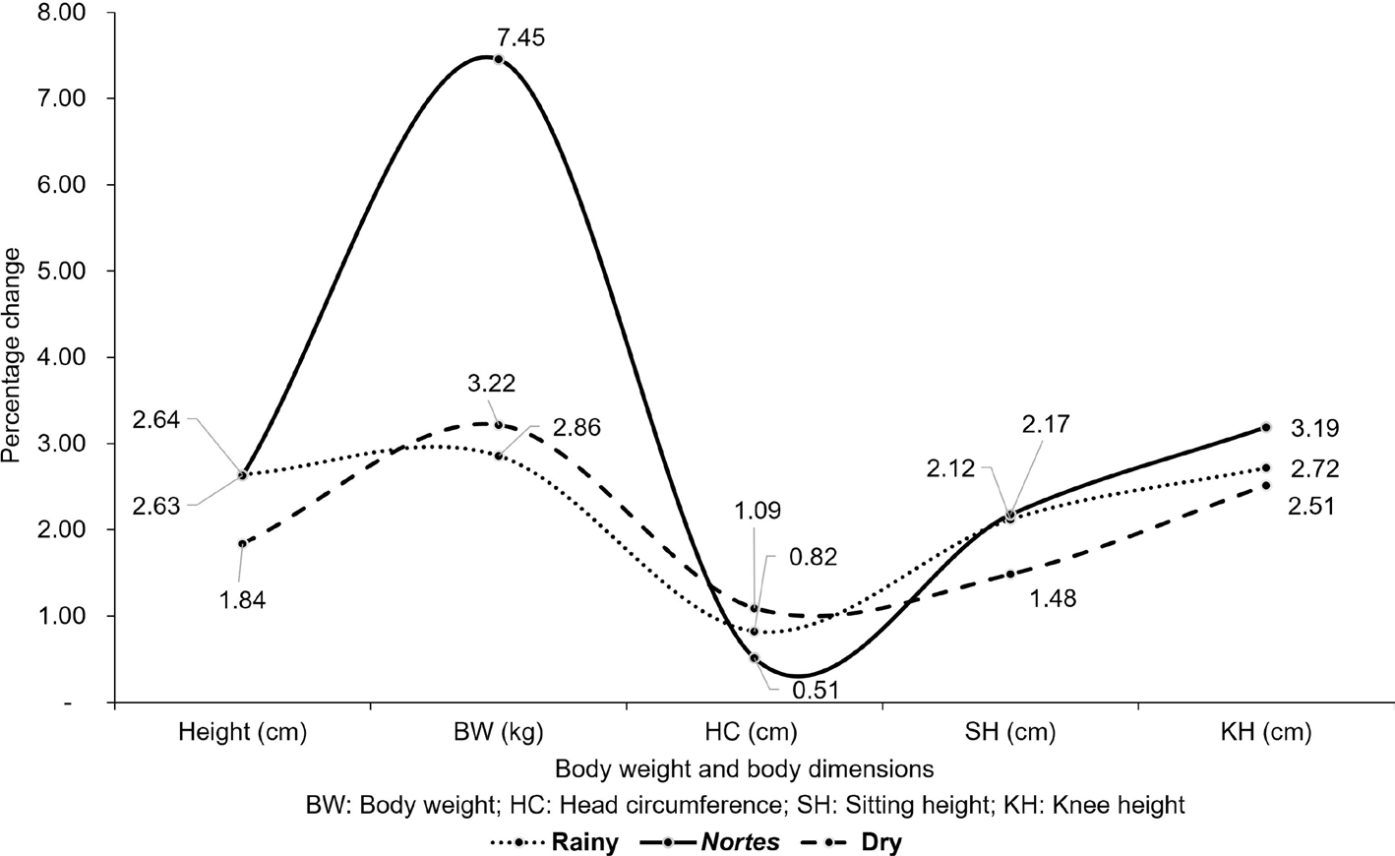
Seasonal variation and differential growth patterns of body weight and body dimensions (percentage change) of 2‐ to 5‐year‐old children (*n* = 31) of Quintana Roo, Yucatan.

Height increment was similar in the rainy and the *nortes* seasons (2.64% and 2.63%, respectively) and was relatively lower in the dry season (1.84%). Increment in BW was highest in the *nortes* (7.45%) compared to the values recorded in the dry (3.22%) and the rainy seasons (2.86%) (Table 3). Seasonal variation of growth was also observed based on the difference in increments from one season to the next. The highest increment of the body dimensions was found in the *nortes* (October to February) compared to other seasons (Figure 2).

**TABLE 3:** Percentage change of anthropometric characteristics (absolute and relative dimensions) across the three seasons and differences between two consecutive seasons in 2‐ to 5‐year‐old children (*n* = 31) of Quintana Roo, Yucatan.

Variables	Percentage change			Paired samples *t*‐test	
	Season 1	Season 2	Season 3		
	June to October (rainy season)	October to February (*nortes* season)	February to June 2022 (dry season)	Seasons 1–2	Seasons 2–3
	Mean (SD)	Mean (SD)	Mean (SD)	*t* (*p*)	*t* (*p*)
Height (cm)	2.64 (1.16)	2.63 (1.16)	1.84 (0.81)	0.05 (0.96)	3.13 **(0.004)**
BW (kg)	2.86 (4.42)	7.45 (5.02)	3.22 (4.06)	3.62 **(0.001)**	4.02 **(< 0.0001)**
HC (cm)	0.82 (0.63)	0.51 (0.53)	1.09 (0.59)	2.13 **(0.04)**	4.55 **(< 0.0001)**
SH (cm)	2.12 (1.49)	2.17 (1.21)	1.48 (0.67)	0.17 (0.87)	2.83 **(0.01)**
SHR (%)	2.13 (1.47)	2.16 (1.21)	−0.34 (1.10)	0.09 (0.92)	8.69 **(< 0.0001)**
KH (cm)	2.72 (1.91)	3.19 (1.47)	2.51 (1.40)	1.13 (0.27)	1.78 (0.08)
KHR (%)	0.08 (1.67)	0.55 (1.28)	0.67 (1.64)	1.28 (0.21)	0.29 (0.77)

*Note:* Bold values = *p* < 0.05.

Abbreviations: BW, body weight; HC, head circumference; KH, knee height; KHR, knee height ratio; SD, standard deviation; SH, sitting height; SHR, sitting height ratio.

The difference in increment between two successive seasons also showed that children had grown well in the *nortes* and the dry compared to those in the rainy season, with significant differences in the values of two consecutive seasons (e.g., increment in the *nortes* versus that in the dry) concerning BW and HC. Mothers and caregivers reported that children fell ill more frequently in the dry season, but the recovery time was less compared to the rainy season when more than one sign and symptom of illness occurred. In this regard, in the dry and the *nortes* seasons, children had greater availability of food, and the duration of illness was shorter, with faster recovery that helped them to grow well physically.

While estimating height‐for‐age and weight‐for‐age *z*‐score‐based nutritional status, it was observed that 17.9%, 13.3%, 12.9%, and 16.1% of children were stunted (low height‐for‐age) in June 2021, October 2021, February 2022, and June 2022, respectively; significant differences in the prevalence of stunting were also found (Table 4). Children were overweight‐for‐age at four different times of measurement: 10.7%, 6.7%, 12.9%, and 17.9% in the dry, rainy, *nortes*, and dry seasons, respectively, showing significant differences (*p* < 0.005) between two successive seasons. A relatively lower prevalence of overweight was observed during the rainy season.

**TABLE 4:** Nutritional status, relative consumption of food items, food production by the households, and signs and symptoms of illness across the three seasons in 2‐ to 5‐year‐old children (*n* = 31) of Quintana Roo, Yucatan.

Variables	A	B	C	D	Paired differences		
					A versus B	B versus C	C versus D
	Mean (SD)	Mean (SD)	Mean (SD)	Mean (SD)			
Low height‐for‐age (%)	17.9	13.3	12.9	16.1	*Χ* ^2^ = 20.66 (*p* < 0.0001)	*Χ* ^2^ = 5.37 (*p* = 0.02)	*Χ* ^2^ = 0.27 (*p* < 0.605)
Overweight‐for‐age (%)	10.7	6.7	12.9	17.9	*Χ* ^2^ = 8.64 (*p* = 0.003)	*Χ* ^2^ = 13.93 (*p* < 0.0001)	*Χ* ^2^ = 22.42 (*p* < 0.0001)
Cereals (%)	29.1 (7.2)	29.9 (6.0)	30.2 (6.2)	*	*t* = 0.66 (*p* = 0.517)	*t* = 0.37 (*p* = 0.712)	NA
Legumes (%)	3.2 (1.7)	3.0 (1.3)	2.9 (1.1)	*	*t* = 0.88 (*p* = 0.384)	*t* = 0.37 (*p* = 0.716)	NA
Vegetables (%)	13.5 (7.7)	13.3 (7.3)	14.2 (6.3)	*	*t* = 0.25 (*p* = 0.805)	*t* = 0.47 (*p* = 0.644)	NA
Fruits (%)	18.7 (9.7)	16.0 (8.7)	13.4 (6.6)	*	*t* = 1.69 (*p* = 0.101)	*t* = 1.81 (*p* = 0.081)	NA
Animal‐origin foods (%)	9.3 (3.5)	11.2 (4.0)	10.8 (2.6)	*	*t* = 2.34 (*p* = 0.026)	*t* = 0.37 (*p* = 0.711)	NA
Dairy products (%)	15.0 (5.5)	13.9 (4.8)	12.1 (5.0)	*	*t* = 1.28 (*p* = 0.211)	*t* = 1.23 (*p* = 0.227)	NA
Fats (%)	0.6 (1.1)	2.2 (2.8)	2.7 (2.3)	*	*t* = 3.88 (***p* = 0.001**)	*t* = 0.68 (*p* = 0.501)	NA
Sugars (%)	10.6 (6.9)	10.5 (7.3)	13.7 (6.3)	*	*t* = 0.09 (*p* = 0.932)	*t* = 2.41 (***p* = 0.023**)	NA
Food production by the households (%)	67.9	53.6	44.4	67.5	*Χ* ^2^ = 5.24 **(*p* = 0.02)**	*Χ* ^2^ = 3.31 (*p* = 0.07)	*Χ* ^2^ = 6.08 **(*p* = 0.01)**
Signs and symptoms of illness (yes) (%)	67.7	45.2	38.7	67.7	*Χ* ^2^ = 1.31 (*p* = 0.252)	*Χ* ^2^ = 3.21 (*p* = 0.073)	*Χ* ^2^ = 2.18 (*p* = 0.14)

*Note:* A: June 2021 (end of the dry season); B: October 2021 (end of the rainy season); C: February 2022 (end of the *nortes* season); D: June 2022 (end of the dry season); cereals: tortilla, rice, bread, etc.; legumes: beans, pulses, etc.; vegetables: available vegetables; fruits: seasonal fruits; animal‐origin foods: meat, fish, egg, etc.; dairy products: milk, yoghurt, cheese, etc.; fats: oil, butter, cream, etc.; sugars: sugary drinks, candys, etc. *data of the dry season (June 2022) were very similar to that of June 2021. Bold values = *p* < 0.05.

Abbreviations: NA, not available; SD, standard deviation.

### 3.3 Children's Eating Habits, Availability of Food Items, and Signs, Symptoms, and Illness

We evaluated children's eating habits through the relative frequency of consumption of eight different food groups. The consumption patterns of cereals (~30%), legumes (~3%), and vegetables (~13.5%) were similar in the three seasons. The relative consumption of animal‐origin foods was slightly higher during the rainy and the *nortes* seasons (~11%) compared to the dry season (~9%), with a statistically significant difference between the dry and rainy seasons (*p* = 0.03). Likewise, from the dry to the *nortes* season, there was a gradual increase in fat consumption (0.6%, 2%, and 3%, respectively), with statistically significant changes between seasons, particularly notable from the dry to the rainy season (*p* < 0.001) (Table 4).

On the other hand, the relative consumption of dairy products decreased marginally from the dry to the *nortes* season through the intermediate rainy season (15%, 14%, and 12%, respectively) without showing a statistically significant difference. A variety of fruits were available in the dry season. Therefore, relative consumption was notably higher (~19%), with a constant decrease in the following seasons (16% and 13%, respectively). Consumption of high‐sugar foods was more significant during the *nortes* season (13.7%), with a statistically significant difference compared to that of the rainy season (11%) (*p* < 0.02) (Table 4). In general, children's eating habits regarding the relative frequency of consumption of different food groups were similar across the seasons, even though we observed some differences due to the availability of food items like fruits and higher consumption of fats in the *nortes* season.

We observed their own production in their backyards (*patio* or *solar*), *milpa*, or plots. The households reported growing fruits and vegetables in the dry season (68%), with a gradual decrease in the rainy (54%) and the *nortes* (44%) seasons; the change from the dry to the rainy season showed a significant difference (*p* = 0.02). Fruit trees predominated in the *patio* or *solar*, with high availability and a great variety of these during the dry season; likewise, to a lesser extent, they also grew vegetables on this site and raised poultry for self‐consumption. In addition, some foods like corn, beans, squash, and pumpkins were grown in the *milpa* and in some plots where the Mexican Government runs the social development program “*Sembrando Vida*” to mitigate HFI. Although household food production served as a food source for the regular household diet, the primary food source was food purchased from local stores.

Mothers and caregivers reported changes in children's health conditions in different seasons. In general, illness of children was more frequent in the dry season (June 2021 and 2022) (~68%), followed by the rainy (45%) and the *nortes* seasons (~39%) in 2021. However, the chi‐square test did not observe significant differences (Table 4). It was interesting to note a statistically significant association between the presence of signs and symptoms of illness and stunting (low height‐for‐age) in the dry season (Spearman rho = −0.4, *p* < 0.03). However, differences in mean values of HAZ and WAZ were not significant (*p* > 0.05) in the rainy season between the group with (a) and without (b) signs and symptoms of illness: HAZ = 0.17 (a), 0.74 (b); WAZ = −0.79 (a), −0.02 (b). Similar results were obtained concerning other seasons (dry and *nortes*). Likewise, the percentage increase in height and BW in the dry, rainy, and *nortes* seasons was not significantly different (*p* > 0.05) between the two groups.

During the interview, the mothers reported that in the dry season, 42% of the children visited the government‐run clinic in the community. That trend decreased in the subsequent rainy (23%) and *nortes* (13%) seasons. Some of the most common signs, symptoms, and illnesses in the dry season were flu (26%), cough (13%), and runny nose (6%). Other less frequent symptoms were fever, diarrhea, and infections. In the rainy season, the most common signs, symptoms, and illnesses, either single or multiple, were flu (16%), fever (16%), vomiting (13%), and gastrointestinal infection (6%), but frequencies were lower than those reported in the dry season. In the *nortes* season, the most common illness reported was flu, which occurred frequently accompanied by other signs or symptoms (cough and fever) (13%), often with diarrhea and vomiting (10%). Another critical aspect was the duration of the three seasons' signs, symptoms, and illnesses. The signs, symptoms, and illnesses lasted longer during the rainy season (7 days, about twice as many days in other seasons).

### 3.4 Repeated Measure ANOVA Test Estimating Seasonality of Physical Growth

The ANOVA (repeated measures) test results (adjusting for age and sex) for height (cm) (values at the end of dry, rainy, *nortes*, and dry seasons in 2021–2022) showed that Box's *M* value (13.91, *p* = 0.31) and Mauchly's test of Sphericity (Mauchly's *W*: 0.52, chi‐square: 4.61, *p* = 0.74) did not violate the assumptions; the variance of the difference between each pair of repeated measures (height) was approximately equal, and thereby sphericity was assumed. Test of between‐subjects effects and the parameter estimates in the separate models for June 2021, October 2021, February 2022, and June 2022 showed that height had a significant positive association with age (*p* < 0.001) after adjusting for sex. Pairwise differences (Bonferroni post hoc test) of mean values of height (cm) (*p* < 0.0001) conformed to the results obtained in paired *t*‐test (Table 2): −2.47 cm (between dry and rainy season), −2.53 cm (between rainy season and *nortes*), and −1.82 (between *nortes* and dry seasons in 2022). We also ran the same analyses for KH (cm) and percentage change (increase) of height across the seasons separately, and similar results were obtained.

## 4 Discussion

The present study reports seasonal variation and differential growth patterns of height, weight, and other body dimensions of preschool children representing the Maya community of Quintana Roo, Yucatan. In the context of the community's relatively homogeneous household SES background, seasonality of all the measured dimensions in children were more associated with the environmental stressor of illness (Molina‐Félix 2022). In general, the increment of BW and other dimensions was relatively higher in the *nortes* season (November to February) and lower in the rainy season (July to October), with intermediate values in the dry season (March to June). The study also observed relatively higher food availability in the dry season, and signs and symptoms of illness did not last as long compared to the rainy and *nortes* seasons.

Children's eating habits regarding the relative frequency of consumption of different food groups were similar across the seasons, even though we observed specific differences due to the availability of food items like fruits in the dry season, an increase in animal protein in the rainy season, and a higher consumption of fats and sugars in the *nortes* season. The most seasonal food was fruit, mainly grown in the *patio* or *solar*; its gradual decrease from the dry season to the *nortes* coincided with an increase in the consumption of fats and sugars, mainly sourced from purchases in local stores.

Other studies carried out in Mayan communities of Yucatan have reported the effects of seasonality on food availability and modifications in household eating patterns, with increases in the purchase of fats and sugars when fruits, vegetables, and meats were scarce (Gurri et al. 2021). Similarly, changes in the structure of the diet, such as increased fats, sugars, and animal protein, have been associated with increases in family income (Popkin 1993). During the rainy and *nortes* seasons, families received monetary support from social programs that may have influenced the increase in the consumption of animal protein, which, due to its high cost compared to other food groups, may be limited during periods of economic scarcity.

Although we observed seasonal changes in children's eating habits and household food production, no associations existed between these and growth. Several studies reported the adverse effects of the rainy season on food availability and growth (Bosha et al. 2019; Guizzo‐Dri et al. 2022; Hillbruner and Egan 2008). However, in our study, there were seasonal changes in some food groups' consumption, but there was no decrease or scarcity of foods across the seasons. Even so, 78% of households in the community reported moderate to severe food insecurity (López‐Moreno 2021), suggesting that there is concern about the availability of desired foods in all seasons. In the present sample, more than 80% of households perceived food insecurity. It was also stated in the report (López‐Moreno 2021) that the Latin American and Caribbean Food Security Scale was not suitable to apply for such a community where homogeneous SES exists in the households along with abundant food (fruits and vegetables) availability in the region that are also grown in the garden (*patio* or *solar*).

Furthermore, while household food production favored dietary diversity, most household food was primarily sourced from purchased food, similar to what has been reported in different rural communities in Yucatan (Castañeda‐Navarrete 2021; Gurri et al. 2021), with constant access throughout the year. Other studies in rural communities in Africa have attributed the absence of associations between subsistence agriculture and inter‐seasonal food patterns to the fact that food acquisition comes from multiple sources, such as own production, purchase, gifts, or exchanges (Ng'endo et al. 2016; Sibhatu and Qaim 2017).

Focusing on each measured variable separately, the percentage of height increase was similar in the rainy (2.64%) and the *nortes* seasons (2.63%) and relatively lower in the dry season (1.84%). The lower increment in the dry season could be influenced by the older age of some participants, having a mean age of 4.56 years at the end of the study when we recorded the final height measurement. The *z*‐score values of height, weight, SH, and KH‐for‐age across the seasons did not have significant differences. From birth to age 6 years, the growth rate progressively decelerates for most children. The BW increase in children of our study was higher in the *nortes* season (7.45%) compared to the values recorded in the dry season (3.22%) and the rainy season (2.86%). It was interesting to observe this tendency, which correlates with a relatively higher consumption of various food groups in the *nortes* season and the more extended duration of illness in the rainy season. The percentage increase of KH was higher in the *nortes* season (3.19%) compared to that in the rainy (2.72%) and the dry seasons (2.51%), and KHR in the dry season (0.67%) compared to that in the *nortes* (0.55%) and the rainy seasons (0.08%). SH and SHR showed a higher percentage of increase in the *nortes* than in other seasons.

Therefore, the *nortes* was the best season for growth. Relatively higher increments of lower leg length (KH) of children compared to trunk (SH) in the *nortes* season could be partially due to the favorable stimuli (increased energy as reflected by BW gain) of the season, a period of superior economic solvency in the community in which, as previously mentioned, families received benefits from government social welfare programs that facilitated access to a greater quantity and quality of food and consequent emotional support. Additionally, in this season, there was a lower incidence of illness in children, which is known to have significant repercussions on growth and was unfavorable during the rainy season.

Regarding this last point, the perception of seasonality may vary for each person depending on their environment. In the study community, during the fieldwork, mothers' knowledge of the seasons was primarily reflected in aspects related to agriculture and their children's health. Many of them identified the *nortes* season as a season in which children needed more care to protect them from changes in weather conditions. One of them said: “Well, in my opinion, the changes of season are, for example, how one time goes from hot to cold, so sometimes I think that if there is such a sudden change in the weather and sometimes, we neglect the children, they get sick… That is the change I see in the environment; they also change.” In this sense, the mother's perception of seasonality related to diseases could have increased the care of children during the *nortes* season and decreased the incidence of diseases. Overall, increases in energy reserve, caregiving, and economic, social, and emotional support appear responsible for the growth patterns observed during this season.

Research shows that the fastest‐growing body dimensions are the most sensitive to environmental influences (reviewed in Bogin and Varela‐Silva 2010). In the present study, the lower leg of the participants was growing relatively faster than their trunk or head, a finding reported by others (Dangour 2001; Frisancho et al. 2001). Osteometric studies in humans have shown that in the segments that make up the leg length, the effects of environmental factors are more significant in the lower segment (the tibia) compared to those in the upper segment (the femur) (Jantz and Jantz 1999). Therefore, the lower segment of the leg (KH) is a particularly sensitive indicator of environmental influences (Dangour 2001; Bogin et al. 2002; Floyd 2008; Vázquez‐Vázquez et al. 2013). In this regard, a longer leg length relative to height is associated with favorable early‐life environments, including nutritional resources, socioeconomic aspects, and good health in general. At the same time, adverse circumstances such as insufficient diet, illnesses, and physical and emotional stress can reduce the growth of the lower leg, both absolute and relative to height, leading to short stature and lower subischial leg length (height minus SH) in adulthood (Bogin and Varela‐Silva 2010).

Finally, the associations between illness and growth showed different interactions. These variables showed an inverse association among signs, symptoms, illness duration, and BW in the rainy season compared to those in the *nortes* season. We further observed that the children who had frequent illnesses in the rainy season had a lower increase in weight gain and another body dimension (overall growth) during the months of this season, which had an effect in the following season that implies a growth trade‐off. Despite having a relatively small sample size, further analysis showed no significant differences in mean values of HAZ and WAZ between the children with and without symptoms of illness in any season (dry, rainy, and *nortes* seasons). However, the frequency of overweight children was higher in the *nortes* when mothers reported relatively less frequent signs and symptoms of illness in children. The relative frequency of consumption of food items across the seasons was similar, although high‐energy foods rich in fats and sugars were consumed more during the *nortes*. These findings could be explained by life history theory, which states that energy is allocated to three main functions of life: growth, maintenance, and reproduction (among children, the third one is absent) (Charnov 1993; Stearns 1992). The energy resources invested in one of these functions are no longer available for use in another; however, excess energy can be stored for future use (McDade et al. 2008).

It has been argued that, even under suitable conditions, the life cycle is replete with competition, i.e., trade‐offs, for resources between growth and maintenance during the early‐life stages (Bogin et al. 2007). Under adverse environmental conditions, such as limited resources and high pathogen exposure, a trade‐off favoring maintenance may happen, leading to growth deficits (Blackwell et al. 2010; Urlacher et al. 2018; Vitzthum et al. 2024). The study's children who used energy to fight infections and recover health after an illness likely had less energy and other nutritional resources for physical growth. Those without illness would have more energy for height growth and could also store energy as fat as a reservoir.

### 4.1 Strengths and Limitations

The present study among preschool children from a rural Maya community of Yucatan is a pioneering one where we analyzed the seasonal variation of growth of body dimensions (absolute and relative to height). The relatively small sample size of the present study was one of the limitations. Furthermore, the sample included children aged 2‐ to 5‐year‐olds. Although growth continues more slowly after 2 years of age compared to the first 2 years of life, within this age range, there are still differential growth rates, both in total size and in body dimensions, between younger and older children, which could differentially influence the growth pattern and body proportionality. The duration of the study could be extended for a longer period. Detailed analysis of the association between child growth and seasonal variation of eating habits, food availability, signs, symptoms, and illness of children needs to be done with a larger sample size.

## 5 Conclusion

Seasonality showed diverse effects on physical growth among preschool children from a Maya community in rural Yucatan, primarily associated with the environmental stressor of illness. BW was most sensitive to environmental influences, followed by KH and SH. Differential increments in leg length and trunk were observed to be relevant in evaluating seasonal growth changes, besides height and weight.

Increment of the dimensions was higher during *nortes*, a period of superior household economic solvency, in which children had greater availability of food (increased energy), and the shorter duration of illness with faster recovery in that season helped them to grow well physically. The percentage increase in height and weight, along with HAZ and WAZ, was not different in the groups of children with and without signs and symptoms of illness.

In general, the study's contribution was to demonstrate the seasonality and differential growth of body dimensions of preschool children, showing minimal impacts of eating habits (relative frequency of consumption of food items) and illness across the seasons in a community with homogeneous household SES.

## Author Contributions

The present report is based on the master's thesis of one of the co‐authors (L.N.M.F.). The authors made equal contributions when writing the manuscript.

## Ethics Statement

The study was conducted according to the guidelines of the Declaration of Helsinki. The research protocol was evaluated and approved by the institutional Bioethics Committee for the Research on Human Beings (COBISH in Spanish acronym) of the Centro de Investigación y de Estudios Avanzados del Instituto Politécnico Nacional (Cinvestav‐IPN).

## Conflicts of Interest

The authors declare no conflicts of interest.

## Data Availability

The authors have nothing to report.
